# Effects of Four Lipid Metabolism-Related Polymorphisms on Body Composition Improvements After 12 Weeks of High-Intensity Interval Training and Dietary Energy Restriction in Overweight/Obese Adult Women: A Pilot Study

**DOI:** 10.3389/fphys.2021.712787

**Published:** 2021-09-01

**Authors:** Omar Andrade-Mayorga, Erik Díaz, Luis A. Salazar

**Affiliations:** ^1^Center of Molecular Biology and Pharmacogenetics, Scientific and Technological Bioresource Nucleus (BIOREN), Universidad de La Frontera, Temuco, Chile; ^2^Department of Preclinical Sciences, Faculty of Medicine, Universidad de La Frontera, Temuco, Chile; ^3^Exercise, Movement and Health Research Group, Universidad de La Frontera, Temuco, Chile

**Keywords:** genetic polymorphisms, exercise, high-intensity interval training, obesity, inter-individual variability, women

## Abstract

**Background:** Polymorphisms in lipid metabolism-related genes have been associated with obesity and body composition, but these have been scarcely described concerning the magnitude of the response to exercise interventions in the overweight/obese population.

**Objective:** To evaluate the association of perilipin 1 (*PLIN1*; rs1052700 and rs2304795), lipoprotein lipase (rs283), and adrenoceptor beta 3 (rs4994) polymorphisms with high and low responders (LoRes) to fat mass reduction after 12 weeks of high-intensity interval training (HIIT) and dietary energy restriction in overweight/obese adult women. In addition, we examined the effect of these genetic variants on body composition changes.

**Methods:** Forty-three unrelated overweight/obese adult women were incorporated and genotyped, of which 30 women (age = 27.4 ± 7.9 years; BMI = 29.9 ± 3.3 kg/m^2^) successfully completed the 12-week supervised HIIT program plus an individually prescribed home hypocaloric diet.

**Results:** An association was observed between the *PLIN1* rs1052700 polymorphism with high and LoRes (*χ*^2^ = 8.138; 2 *df*; *p* = 0.01). Moreover, after the intervention, the carriers of TT genotype of *PLIN1* rs1052700 as compared to AA and AT showed a greater reduction in absolute fat mass (Δ: −5.1 ± 1.8 vs. − 1.8 ± 1.4 vs. − 2.1 ± 2.3 kg; *p* = 0.04). The effect size of this fat mass reduction between TT and AT genotypes was a mean difference of −3.01 kg [95%IC − 4.88– − 1.1], and between TT and AA genotypes was −3.29 kg [95%IC − 4.86– − 1.65]. No differences were observed for other polymorphisms investigated.

**Conclusion:** These results suggest that the rs1052700 (14995A>T) polymorphism of the *PLIN1* gene is associated with a differential response to fat mass reduction after a 12-week intervention in overweight/obese adult women. In addition, women with the TT genotype of this genetic variant showed greater changes in fat mass than AA and AT genotypes. However, further studies are needed to confirm these findings.

## Introduction

Obesity and overweight have become major global health problems over the past few decades (Heymsfield and Wadden, 2017) because the expansion of white adipose tissue (adipocyte hypertrophy) and visceral adiposity leads to metabolic dysregulation as a result of their associated pro-inflammatory phenotype (Vazquez-Carrera, 2016; Hepler and Gupta, 2017). Importantly, there is a global gender disparity in obesity, with a higher prevalence in women than men in all world regions (Ahmad et al., 2016). Specifically, obese women have a higher risk of developing type 2 diabetes (T2D), and these women have a 44% greater risk of cardiovascular disease than men (Hu, 2003; Peters et al., 2014), as well as a higher risk of morbidity and mortality from T2D, cardiovascular diseases, cancer, and other obesity-related conditions (Ahmad et al., 2016). Behavioral interventions, such as exercise and nutrition, have been essential in managing obesity and overweight because of their contribution to reducing fat and body mass (Petridou et al., 2019). However, studies reporting individual responses to exercise have shown a wide range of responses to the interventions rather than a similar response (Bouchard and Rankinen, 2001; King et al., 2008; Scharhag-Rosenberger et al., 2012; Bonafiglia et al., 2016; Gurd et al., 2016; Parr et al., 2016; Alvarez et al., 2017; de Lannoy et al., 2017; Sparks, 2017; Williamson et al., 2017; Chrzanowski-Smith et al., 2019; Ross et al., 2019). Recent evidence shows that genetic and epigenetic factors could contribute significantly to the inter-individual variability in response to exercise interventions (Sparks, 2017; Williamson et al., 2017; Hagstrom and Denham, 2018). Regarding genetic factors, although some single-nucleotide polymorphisms (SNPs) in lipid metabolism-related genes have previously been associated with obesity and body composition, they have been scarcely described concerning the magnitude of the response to exercise interventions in the overweight/obese population. In the present study, four candidate gene polymorphisms related to lipid metabolism and fat mass response after exercise training were studied as: rs2304795 and rs1052700 variants of the perilipin 1 (*PLIN1*) gene, rs283 variant of the lipoprotein lipase (*LPL*) gene, and rs4994 variant of the Adrenoceptor beta 3 (*ADRB3*) gene. In this sense, *PLIN1* gene has recently emerged as a candidate gene to explain part of the inter-individual differences in cardiovascular and metabolic risk factors (Jenkins et al., 2010). The different isoforms of the perilipin protein encoded by the *PLIN* gene are proteins that coat lipid droplets and regulate intracellular lipolysis (Bickel et al., 2009). The *LPL* gene encodes the *LPL* enzyme, which plays a fundamental role in lipid metabolism since it is the rate-limiting enzyme for the hydrolysis of triglycerides (TG). For this reason, it has been related to plasma lipid levels and the development of obesity (Xie et al., 2010; Gao et al., 2015). In addition, an association has been found between the rs283 polymorphism of the *LPL* gene and blood lipids levels, with higher levels of high-density lipoprotein-cholesterol in the carriers of GG genotype (Sarzynski et al., 2011). This same GG genotype was more sensitive to reducing fat mass, insulin resistance, and plasma triglyceride level induced by 4 weeks of supervised physical exercise in Asian adolescents with obesity (Gao et al., 2015). *ADRB3* gene encoded the human β3-adrenergic receptor (β3AR), expressed mainly in adipose tissue, plays a role in determining the basal metabolic rate (BMR) through its ability to stimulate lipolysis and thermogenesis (Kahara et al., 2002). Therefore, a dysfunction in the β3AR may increase the risk of developing obesity and insulin resistance (Zhan and Ho, 2005). The rs4994 (Trp64Arg) polymorphism of the *ADRB3* gene has shown a tendency to have a low resting metabolic rate, abdominal obesity, insulin resistance, and the development of T2D. Therefore, it has been widely studied as a potential genetic factor associated with the development of obesity but with inconclusive results (Nakashima et al., 2013). A study in healthy young Japanese men found that subjects carrying the Arg64 allele of the *ADRB3* gene have a reduction in fatty acid oxidation at rest and during acute physical exercise (Morita et al., 2009). Thus, the present study aimed to evaluate the association of lipid metabolism-related polymorphisms (*LPL* rs283, *PLIN1* rs2304795, *PLIN1* rs1052700, and *ADRB3* rs4994) with a high or low response to fat mass reduction after 12 weeks of high-intensity interval training (HIIT) in overweight/obese adult women. Additionally, we examine whether body composition changes are influenced by these polymorphisms.

## Materials and Methods

### Study Design and Participants

A group of unrelated adult overweight and obese women was studied. Participants were recruited from the community and referred by a physician to the supervised exercise program in our research center. The study was conducted following the Declaration of Helsinki Ethical Principles for Medical Research involving human subjects. Ethical approval for the study was provided by the Scientific Ethics Committee based at Universidad de La Frontera (Study Protocol N°112/16). All volunteers received information about the protocol and provided written consent before the beginning of the study.

**Figure 1:**
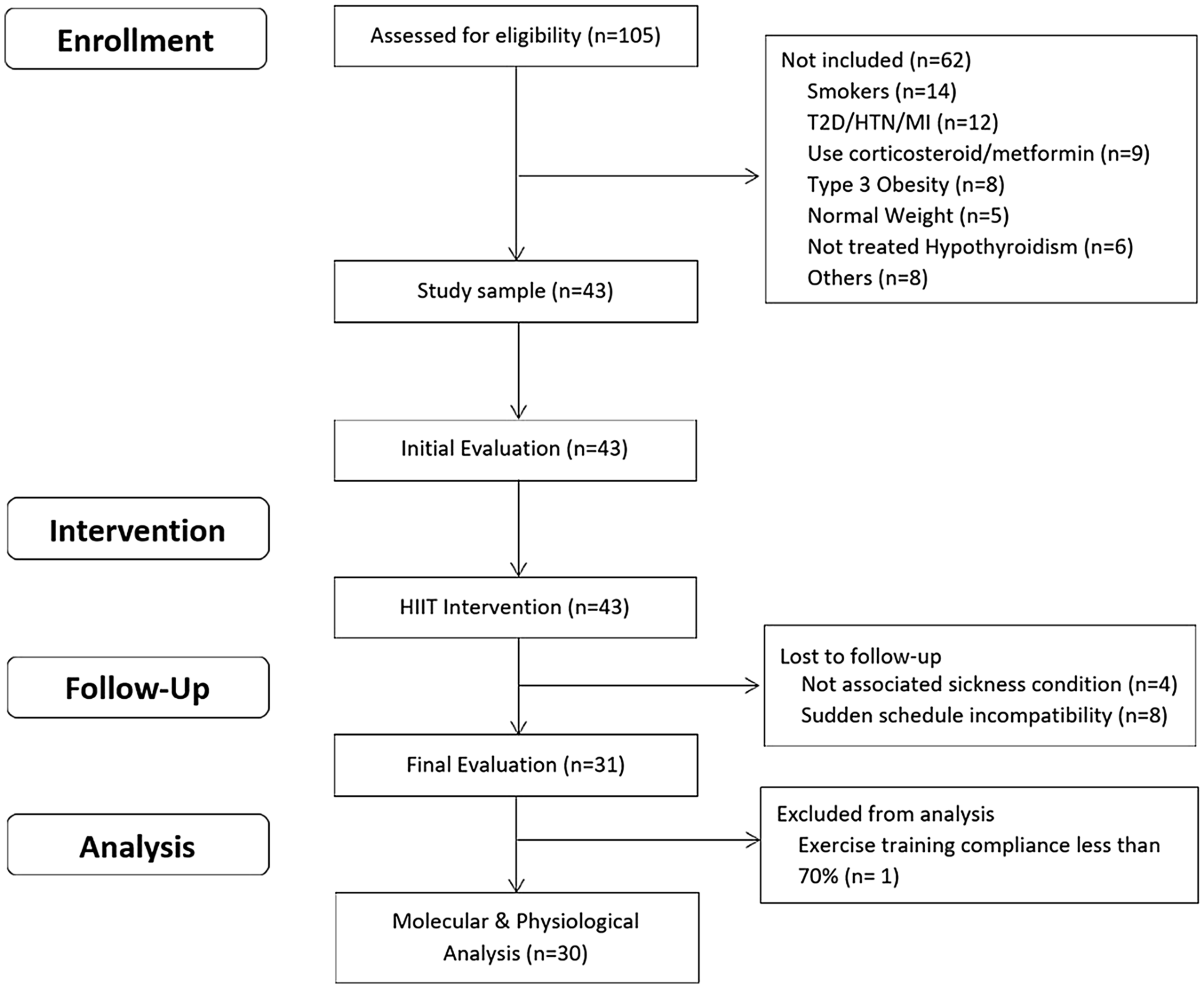
Study design.

**Figure 2:**
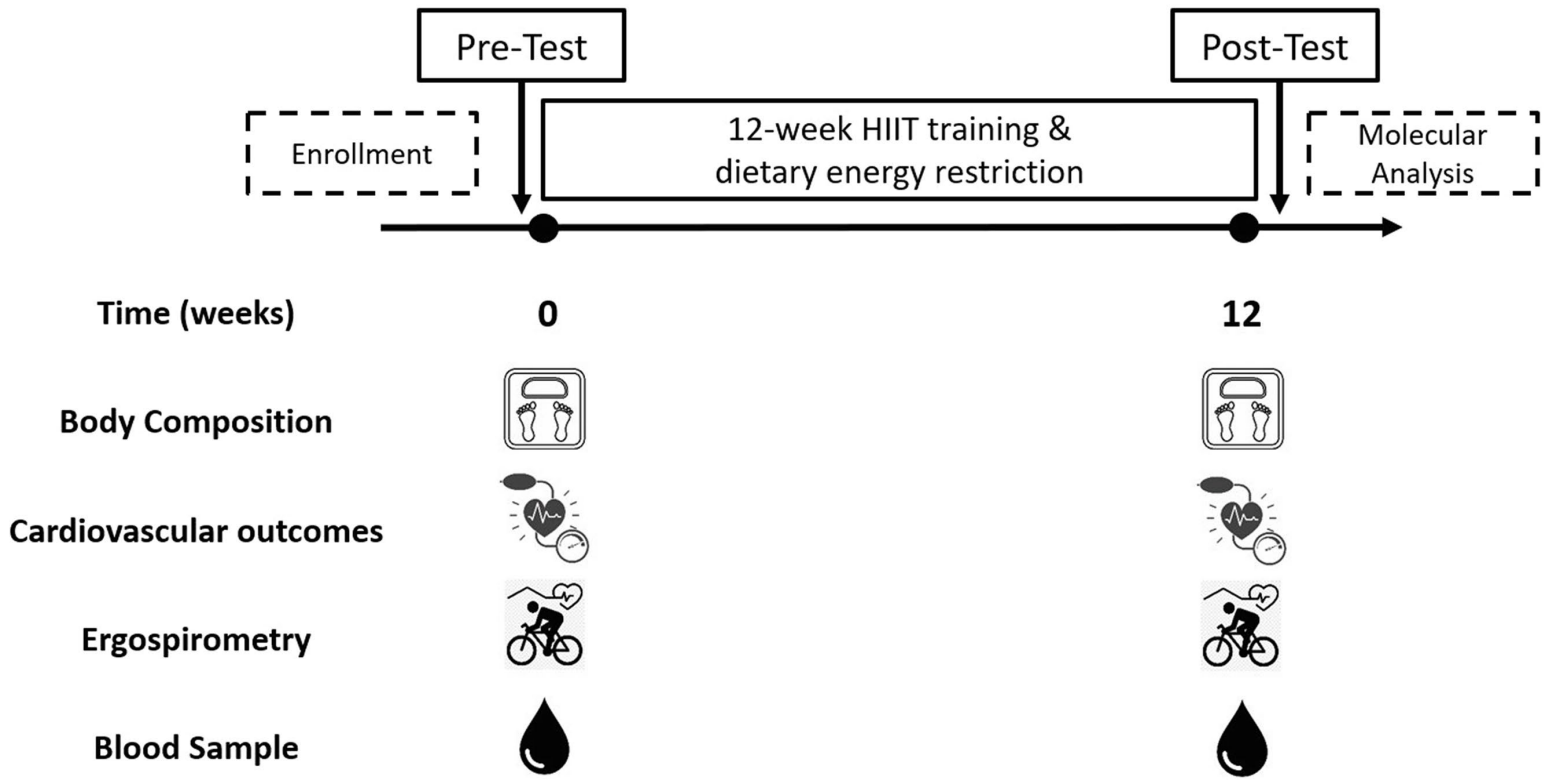
Study protocol. Pre-test: initial assessments; Post-test: final assessments.

The inclusion criteria were as: a) unrelated women aged 18–45 years; b) diagnosed with overweight or type 1 or 2 obesity [Body mass index (BMI) between 25 and 39.9 kg/m^2^]; c) untrained (not involved in regular physical activity or exercise program during the previous 6 months); d) pre-menopausal women; and e) previously screened by a physician. The exclusion criteria were as: a) previously diagnosed diseases, such as diabetes mellitus, hypertension, myocardial infarction, and class III obesity; b) receiving pharmacologic corticoids, metformin, or other drugs that may affect metabolism; c) smoking habit; d) history of bariatric surgery; e) untreated hypothyroidism; and f) skeletal muscle disabilities or a specific indication to avoid exercise by medical reasons. A minimum of 70% (26/36 sessions) attendance at the exercise program was required for study participants to be included in the final statistical analyses. A structured medical history record and physical examination were performed on 105 adult women for enrolment purposes. Forty-three subjects met all the inclusion criteria and were finally studied. This study used a non-probabilistic sample aiming to separate high and low responders (LoRes) to a 12-week exercise program. Subjects who successfully completed the 12 weeks of intervention (*N* = 30) were classified as high or LoRes according to their greater or lesser reduction in fat mass, similar to previous studies that have also used clinical cutoff points for this classification (Milagro et al., 2013; Parr et al., 2016). Thus, high responders (HiRes) to fat mass reduction after the 12-week intervention were those individuals who were able to lose ≥10% of initial absolute fat mass (i.e., kilograms), and LoRes were individuals who lost <10%. Body composition, endurance performance, resting blood pressure, fasting glucose, and insulin were assessed before and after the 12-week follow-up. All the molecular analyses were performed after the 12-week intervention. The study design is shown in Figure 1, and the study protocol is shown in Figure 2.

### Body Composition Assessment and Cardiovascular Measurements

The initial assessment was carried out to record socio-demographic, physical, and physiological characteristics. Body mass, absolute fat mass, and fat-free mass (FFM) were assessed with the subject barefoot, wearing underclothes, and no metal objects, using a Tanita^™^ foot-foot bioelectrical impedance analyzer (BIA) (Tanita Corporation, model BC-541, Japan). Its prediction formula has been previously validated against a four-compartment model, showing high reproducibility and a residual standard deviation of 3.3% for body fat in women (Jebb et al., 2000). Stature was measured without shoes to the nearest mm with a stadiometer (Seca model 213, Hamburg, Germany). BMI was calculated using the formula body mass divided by stature squared (kilograms per square meter). The systolic and diastolic blood pressures were determined using an automatic monitor (Omron HEM-7114; Omron Healthcare) in duplicate and after 15 min of complete rest with the subjects in a supine position.

### Endurance Performance Assessment

Endurance performance was assessed 1 week before and after the 12-week intervention during an incremental exercise test designed to obtain peak oxygen consumption (VO_2_peak). In brief, the VO_2_peak test consisted of free-wheel pedaling for 2 min at 70–80 RPM on a cycle ergometer (Lode Corival, Groningen, The Netherlands), followed by an initial 50 watts load for 2 min and 25 watts increments every 2 min until the participant reached volitional fatigue. Gas exchange was collected throughout the test using an indirect calorimeter/ergospirometer system (Ultima CPX^™^ metabolic system, Medgraphics, Minnesota, United States), calibrated (gas and volume) before the exercise test. Measurements performed were the peak power output (PPO), anaerobic threshold (AT), respiratory exchange ratio (RER), peak oxygen pulse (O_2_ pulse; VO_2_peak/HRmax during the exercise test), ventilation (VE), and respiratory rate (RR). Heart rate (HR) was monitored with a continuous telemetric HR sensor (Polar model V800, Finland) throughout the whole test.

### Exercise Training Intervention

Participants performed a 12-week supervised HIIT program, with training sessions three times a week on non-consecutive days. The exercise session consisted of 1-min cycling at a high intensity (workload during each interval was set to achieve muscle failure at the end of 1-min exercise period and reaching ~85–100% maximal heart rate obtained during the incremental exercise test), followed by a 2-min inactive resting period (sitting on the cycle ergometer), and repeated 10 times (1x2x10 protocol; 1:2:10 to work: rest: repetitions, respectively). In summary, the total duration of one session of the 1x2x10 protocol was 30 min, with 10 min of effective exercise training, and without a warm-up or cool-down period. All exercise sessions were individually supervised to achieve muscle fatigue at each exercise interval as the primary indicator of intensity together with a continuous heart rate monitoring (Polar V800, PolarTM, Finland) in order to supervise that the chronotropic response was the expected according to previously found with the same HIIT exercise protocol (Andrade-Mayorga et al., 2020). Load progression was defined as the gradual increase in workload developed when the subject failed to reach muscle failure at the end of the 60-s exercise interval, monitored individually on a series-by-series and session-by-session basis. Thus, the load progression increased in parallel to the increment in the work capacity of each individual.

### Dietary Assessment and Hypocaloric Diet

Participants were instructed to follow an individually designed hypocaloric diet [75% of estimated energy requirements (EERs)], equivalent to 1354 ± 114.5 kcal/day, throughout the 12-week study period to control the dietary intake. A 24-h diet recall and a modified food choice questionnaire (7 days) were applied at baseline (Salvador Castell et al., 2015). Total energy expenditure (TEE) was estimated using the factorial method based on their reported daily physical activity (FAO/WHO/UNU, 2004; Levine, 2005). In brief, BMR was estimated using the Mifflin-St. Jeor equation (Cancello et al., 2018), additional caloric requirements were determined based on each subject’s physical activity level (PAL), which were used to calculate the PAL index, TEE, and the 75% EER. Subjects had individual monthly meetings with the nutritionist during the 3-month intervention to encourage compliance. Dietary compliance was assessed using an instrument adapted from the Perceived Self-Regulatory Success in Dieting Scale (Meule et al., 2012), where each subject’s perceived adherence was quantified using a five-point Likert scale. The nutritional intervention excluded the use of nutritional supplements.

### Blood Analyses

Blood samples (5 ml) were collected to analyze plasma glucose and insulin in the early morning after 12 h overnight fasting, and they were immediately placed on ice and centrifuged at 3000 rpm for 15 min at −4°C. Plasma samples were directly transferred to pre-chilled microtubes and stored at −20°C for later analysis. Fasting plasma glucose was analyzed by enzymatic colorimetric methods using an auto-analyzer (Wiener Metrolab 2300, Wiener Lab, Argentina). The fasting insulin was analyzed by ELISA using the Human Insulin ELISA Kit (Catalog # KAQ1251, Invitrogen, Thermo Fisher Scientific Inc., Waltham, MA, United States).

### DNA Genotyping

Genomic DNA was extracted from blood leukocytes by optimized salting out procedure (Salazar et al., 1998). Genotyping of *LPL* rs283, *PLIN1* rs2304795, *PLIN1* rs1052700 (14995A>T), and *ADRB3* rs4994 polymorphisms were performed by real-time polymerase chain reaction (qPCR), using TaqMan^®^ SNP Genotyping Assays (Life Technologies, CA, United States). PCR assays contained 12.5 μl of TaqMan^®^ Genotyping Master Mix (2X; Life Technologies CA, United States), 1.25 μl of TaqMan^®^ SNP Genotyping Assay (20X; catalog numbers: 4351379, 4351379, 4351379, and 4351379), and 2 μl of DNA (25 ng) diluted in nuclease-free water. The thermal cycling protocol was initiated with a cycle for 10 min at 95°C and followed by 40 cycles at 95°C for 15 s and 60°C for 1 min using standard conditions for a real-time system (Life Technologies). Genotyping was performed using the allelic discrimination plot issued after PCR amplification in the StepOne software v. 2.2 (Life Technologies). No template controls were included per triplicate in each genotyping experiment plate. Genotyping was randomly repeated on 20% of the samples for quality control purposes without finding differences.

### Statistical Analysis

GraphPad Prism statistical software 7.0 (San Diego, CA, United States) was used. Chi-square test (*χ*^2^) was used to analyze differences in genotype distribution and allelic frequencies and verify Hardy–Weinberg equilibrium. The normal distribution of all the variables was tested using the D’Agostino-Pearson test. All the continuous variables were expressed as mean ± standard deviation (SD). The differences between quantitative variables were analyzed by paired *t*-test for paired data (intra-group differences before and after intervention) or unpaired *t*-test for independent groups (between-groups difference within time points). Cumming estimation plots, which show individual values, means, and effect size with a 95% CI, were developed using Estimation Statistics for Data Visualization (Ho et al., 2019). Kruskal-Wallis test was used to compare the body composition changes among the different genotypes. Differences between two specific genotypes were evaluated with the Mann-Whitney *U* test. The level of significance used in all the comparisons was *p* < 0.05.

## Results

Forty-three unrelated overweight/obese adult women volunteered for the study, of which 30 women (age = 27.4 ± 7.9 yrs; BMI = 29.9 ± 3.3 kg/m^2^) successfully completed the 12-week intervention and were finally analyzed. All participants well tolerated the exercise program, and there were no injuries during the intervention. Demographic, physical, and physiological variables measured before and after the intervention of the HiRes and LoRes groups are presented in Table 1. At baseline, the LoRes and HiRes groups are similar in age (27.6 ± 8.6 vs. 27.2 ± 7.2 years; *p* = 0.897), PAL index (1.38 ± 0.08 vs. 1.41 ± 0.09; *p* = 0.143), BMR (1,283 ± 55.7 vs. 1,314 ± 48.1 kcal/day; *p* = 0.437), TEE (1778 ± 159.9 vs. 1852 ± 133.4 kcal/day; *p* = 0.140), and 75% EER (1,333 ± 119.9 vs. 1,389 ± 100.1 kcal/day; p = 0.140; Table 1). Diet compliance measured after the intervention was similar between groups (3.3 ± 0.5 vs. 3.6 ± 0.5 points; *p* = 0.3211; Table 1). The prevalence for high (HiRes) and low (LoRes) responders to absolute fat mass reduction was 33% (*n* = 11) and 66% (n = 19), respectively. The intervention applied in this study effectively improved several variables, showing significant changes in both groups (LoRes and HiRes) in reducing body mass, absolute fat mass (kg), % body fat, and BMI. In addition, the 12-week intervention was effective in increasing VO_2_peak and VO_2_peak relative to lean mass (VO_2_peak/FFM) similarly in both groups (LoRes and HiRes; Table 1). FFM did not change post-intervention in either group (Table 1).

**Table 1:** Demographic, physical, and physiologic variables measured before and after intervention by low (LoRes) and high (HiRes) responders to fat mass reduction after the 12-week intervention.

	Low Responders (*n* = 19)			High Responders (*n* = 11)			Intra-group *p*-value (pre vs. post)^*^		Between groups *p*-value (within same time point)^#^		*P*-value Δ^¥^
	Pre	Post	Δ	Pre	Post	Δ	LoRes	HiRes	Pre	Post	
Age (years)	27.6 ± 8.6	–	–	27.2 ± 7.2	–	–	–	–	NS	–	–
PAL index	1.38 ± 0.08	–	–	1.41 ± 0.09	–	–	–	–	NS	–	–
BMR (kcal/day)	1,283 ± 55.7	–	–	1,314 ± 48.1	–	–	–	–	NS	–	–
TEE (kcal/day)	1778 ± 159.9	–	–	1852 ± 133.4	–	–	–	–	NS	–	–
75% EER (kcal/day)	1,333 ± 119.9	–	–	1,389 ± 100.1	–	–	–	–	NS	–	–
Diet compliance (pts)	–	3.3 ± 0.5	–	–	3.6 ± 0.5	–	–	–	–	NS	–
Body weight (kg)	77.2 ± 9.8	76.2 ± 9.7^*^	−1.0 ± 0.9	76.1 ± 11.1	71.3 ± 9.9^*^	−4.8 ± 2.7^¥^	**0.0004**	**0.0001**	NS	NS	**<0.0001**
Fat mass (kg)	31.6 ± 6.9	30.5 ± 6.7^*^	−1.1 ± 0.9	29.8 ± 8	25.2 ± 6.9^*^^,^^#^	−4.7 ± 1.6^¥^	**<0.0001**	**<0.0001**	NS	**0.04**	**<0.0001**
% Body fat (%)	40.5 ± 4	39.6 ± 3.9^*^	−0.9 ± 0.9	38.6 ± 4.9	34.7 ± 5.1^*^^,^^#^	−3.8 ± 1.1^¥^	**0.0003**	**<0.0001**	NS	**0.0068**	**<0.0001**
BMI (kg/m^2^)	30.6 ± 3.3	30.2 ± 3.3^*^	−0.4 ± 0.4	28.8 ± 3	27 ± 2.8^*^^,^^#^	−1.8 ± 1.0^¥^	**0.0003**	**0.0001**	NS	**0.01**	**<0.0001**
FFM (kg)	45.6 ± 3.5	45.8 ± 3.5	0.1 ± 0.7	46.3 ± 3.5	46.2 ± 3.7	−0.1 ± 1.8	NS	NS	NS	NS	NS
VO2peak (ml/min)	1750 ± 230	2051 ± 332^*^	301.2 ± 240.2	1888 ± 266	2,169 ± 322^*^	281.1 ± 177.3	**<0.0001**	**0.0004**	NS	NS	NS
VO2peak/FFM (ml kg^−1^ min^−1^)	38.3 ± 3.6	44.7 ± 5.6^*^	6.4 ± 5.0	40.7 ± 3.9	46.9 ± 5.4	6.2 ± 4.6	**<0.0001**	**0.001**	NS	NS	NS

* intra-group difference pre- vs. post-intervention.

# between-groups difference within same time points.

¥ difference between average delta values.

Data are presented as mean ± *SD*. LoRes, low responders; HiRes, high responders; PAL, physical activity level; BMR, basal metabolic rate; TEE, total energy expenditure; EER: estimated energy requirement; BMI, body mass index; FFM, fat-free mass; and VO_2_peak, maximal oxygen consumption. Bold values denote significant differences.

**Figure 3:**
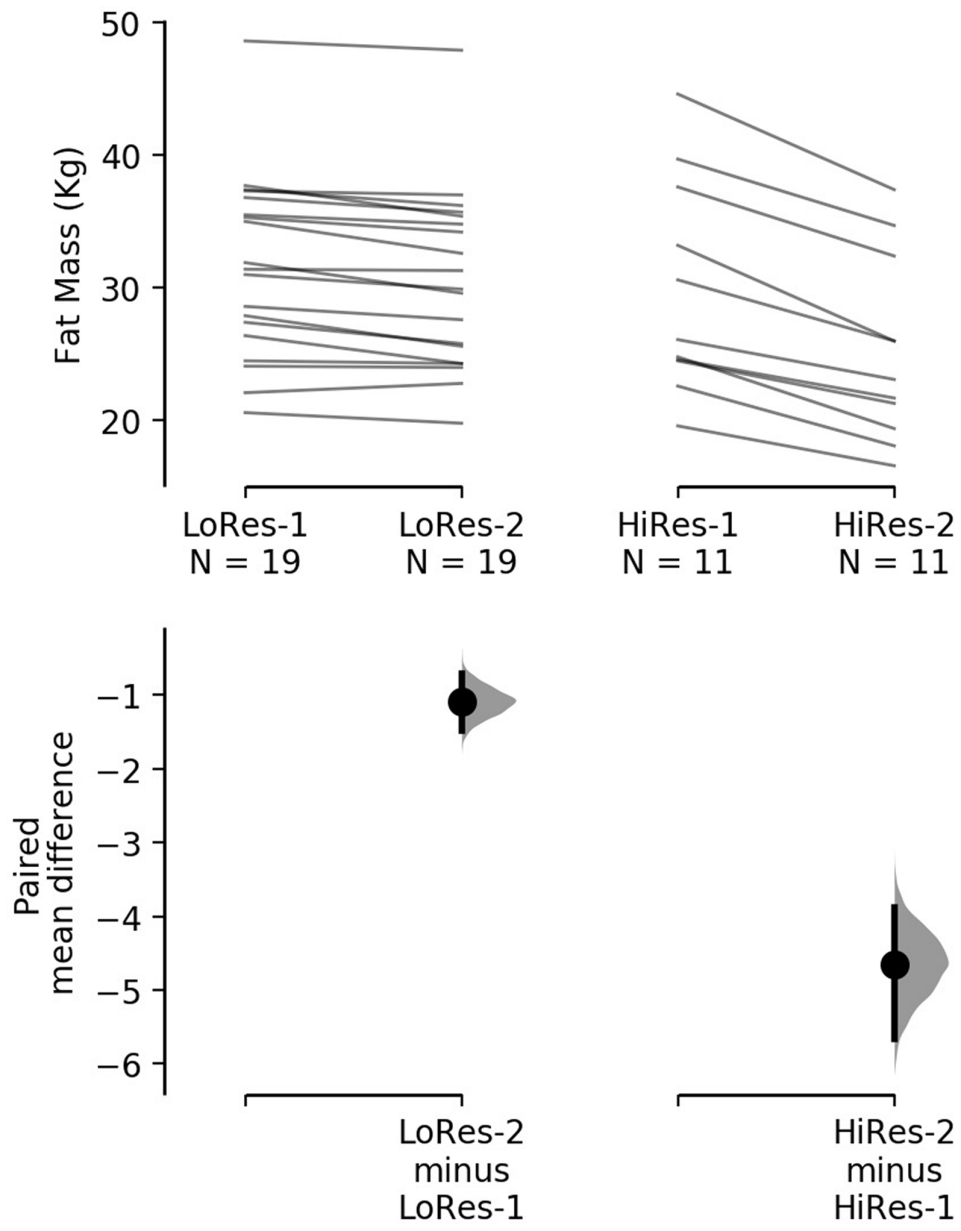
Cumming plot with individual responses to decrease absolute fat mass after the 12-week intervention by low (left) and high (right) responders. The raw data (kg) are plotted on the upper axes; each paired set of observations is connected by a line. On the lower axes, each paired mean difference is plotted as a bootstrap sampling distribution. Mean differences are depicted as dots; 95% confidence intervals are indicated by the ends of the vertical error bars. LoRes-1, initial assessment of low responders; LoRes-2, final assessment of low responders; HiRes-1, initial assessment of high responders; and HiRes-2, final assessment of high responders.

However, it is noteworthy that the reduction in absolute fat mass (kg) in HiRes was 18.3% (29.8 ± 8 vs. 25.2 ± 6.9 kg; *p*<0.0001) and LoRes was 3.6% (31.6 ± 6.9 vs. 30.5 ± 6.7 kg; *p*<0.0001; Table 1). The effect size of this reduction in absolute fat mass was −4.65 kg [95.0%CI − 5.66– − 3.88] for the HiRes group and was −1.09 kg [95.0%CI − 1.49– − 0.705] for the LoRes group (Figure 3). When comparing the magnitude of change (Δ) in absolute fat mass between the LoRes and HiRes groups, this difference was statistically significant (−1.1 ± 0.9 vs. −4.7 ± 1.6 kg; *p* < 0.0001; Table 1). Similarly, differences were found between LoRes and HiRes in the magnitude of changes (Δ) in body mass (−1.0 ± 0.9 vs. −4.8 ± 2.7 kg; *p* < 0.0001), % body fat (−0.9 ± 0.9 vs. −3.8 ± 1.1%; *p* < 0.0001), and BMI (−0.4 ± 0.4 vs. − 1.8 ± 1.0 kg/m^2^; *p* = 0.01) following the 12-week intervention (Table 1).

Physical performance, physiological, and cardiovascular variables measured pre- and post-12-week intervention of the LoRes and HiRes are listed in Table 2. Concerning performance indicator variables during the incremental exercise test, there were improvements in most variables measured post-intervention, but no differences between LoRes and HiRes groups. There were increases in both groups in peak oxygen pulse (Peak O_2_pulse; LoRes: 10.2 ± 1.6 vs. 11.4 ± 1.6 ml/min; *p* = 0.0002/ HiRes: 10.4 ± 1.2 vs. 11.8 ± 1. 7 ml/min; *p* = 0.0002), (PPO; LoRes: 126.3 ± 17.6 vs. 156.6 ± 24.8 W; *p* = 0.0001/HiRes: 131.8 ± 16.2 vs. 165.9 ± 23. 1 W; *p* = 0.0002), and anaerobic threshold (AT; LoRes: 57.4 ± 11.7 vs. 69.1 ± 16.6 W; *p* = 0.0068/ HiRes: 59.1 ± 12.6 vs. 70.5 ± 10.1 W; *p* = 0.03). In addition, in the LoRes group, maximum ventilation (VEmax; 63.5 ± 9.7 vs. 77.4 ± 14.1 ml/min; *p* = 0.001) and maximum respiratory rate (RRmax; 36.7 ± 6.9 vs. 40.0 ± 5.4 cycles/min; *p* = 0.01) increased (Table 2). In relation to cardiovascular variables at rest, there was a decrease of diastolic blood pressure (LoRes: 73.2 ± 8.7 vs. 74.2 ± 5.2 mmHg; *p* = 0.001/HiRes: 74.2 ± 5.2 vs. 70.6 ± 5.8 mmHg; *p* = 0. 005) and mean blood pressure (LoRes: 85.7 ± 7.9 vs. 80.7 ± 7.3 mmHg; *p* = 0.001/HiRes: 85.8 ± 6.2 vs. 82.4 ± 6.6 mmHg; *p* = 0.002) in LoRes and HiRes groups, and a reduction of systolic blood pressure only in the LoRes group (110.9 ± 8.4 vs. 104.7 ± 9.1 mmHg; *p* = 0.03; Table 2). Importantly, all these changes occurred, although none of the study participants had arterial hypertension. About metabolic parameters, no differences were found in fasting glycemia, but there was an inter-group difference in blood fasting insulin after the intervention, where the LoRes group showed higher values than the HiRes group (11.9 ± 2.3 vs. 9.4 ± 2.2.2 μU/dl; *p* = 0.04; Table 2).

**Table 2:** Physical performance, cardiovascular, and metabolic variables measured before and after intervention by low (LoRes) and high (HiRes) responders to fat mass reduction after the 12-week intervention.

	Low Responders (*n* = 19)			High Responders (*n* = 11)			Intra-groups *p*-value (pre vs. post)		Between-groups *P*-value (within time point)		*P*-value Δ^¥^
	Pre	Post	Δ	Pre	Post	Δ	LoRes	HiRes	Pre	Post	
Peak O2 Pulse (ml/min)	10.2 ± 1.6	11.4 ± 1.6^*^	1.2 ± 1.1	10.4 ± 1.2	11.8 ± 1.7^*^	1.4 ± 0.8	**0.0002**	**0.0002**	NS	NS	NS
PPO (W)	126.3 ± 17.6	156.6 ± 24.8^*^	30.3 ± 13.4	131.8 ± 16.2	165.9 ± 23.1^*^	34.1 ± 20.2	**0.0001**	**0.0002**	NS	NS	NS
AT (W)	57.4 ± 11.7	69.1 ± 16.6^*^	10.5 ± 15.2	59.1 ± 12.6	70.5 ± 10.1^*^	11.4 ± 13.1	**0.0068**	**0.03**	NS	NS	NS
VEmax (ml/min)	63.5 ± 9.7	77.4 ± 14.1^*^	13.9 ± 13.0	71.3 ± 21.3	76.9 ± 13.5	5.6 ± 16.6	**0.001**	NS	NS	NS	NS
RRmax (breath/min)	36.7 ± 6.9	40.0 ± 5.4^*^	3.3 ± 5.6	37.4 ± 10.6	37.8 ± 5.6	0.5 ± 6.6	**0.01**	NS	NS	NS	NS
RERmax	1.27 ± 0.1	1.28 ± 0.1	0.01 ± 0.15	1.28 ± 0.1	1.27 ± 0.1	0.02 ± 0.17	NS	NS	NS	NS	NS
Systolic BP (mmHg)	110.9 ± 8.4	104.7 ± 9.1^*^	−6.7 ± 4.5	108.9 ± 9.4	106 ± 9.8	−3.4 ± 2.1^*^	**0.03**	NS	NS	NS	**0.03**
Diastolic BP (mmHg)	73.2 ± 8.7	68.7 ± 7.6^*^	−5.1 ± 6.8	74.2 ± 5.2	70.6 ± 5.8^*^	−4.1 ± 3.7	**0.001**	**0.005**	NS	NS	NS
Mean BP (mmHg)	85.7 ± 7.9	80.7 ± 7.3^*^	−5.6 ± 5.4	85.8 ± 6.2	82.4 ± 6.6^*^	−3.9 ± 2.8	**0.001**	**0.002**	NS	NS	NS
Pulse pressure (mmHg)	37.7 ± 7.2	36.1 ± 7.8	−1.6 ± 6.1	34.7 ± 6.4	35.4 ± 7	0.7 ± 3.2	NS	NS	NS	NS	NS
Fasting glucose (mg/dl)	91.2 ± 10.1	89.4 ± 9.3	−1.8 ± 7.7	89.5 ± 7.2	89.1 ± 7.5	−0.4 ± 6.7	NS	NS	NS	NS	NS
Fasting insulin (μU/dl)	11.9 ± 2.3	11.9 ± 1.6	−5.0 ± 6.8	10.7 ± 2.6	9.4 ± 2.3^*^	−6.4 ± 6.2	NS	NS	NS	**0.04**	NS

* intra-group difference pre- vs. post-intervention.

# between-groups difference within same time point.

¥ difference between average delta values.

Data are presented as mean ± *SD*. O_2_ pulse, oxygen pulse; PPO, peak power output; AT, anaerobic threshold; VEmax, peak ventilation; RRmax, peak respiratory rate; RERmax, peak respiratory exchange ratio; and BP, blood pressure. Bold values denote significant differences.

Genotypes distribution and relative frequency of alleles for *PLIN1* rs1052700, *PLIN1* rs2304795, *LPL* rs283, and *ADRB3* rs4994 gene polymorphisms are shown in Table 3. All polymorphisms evaluated were in Hardy-Weinberg equilibrium (Table 3).

**Table 3:** Genotype distribution and relative allelic frequencies for studied lipid metabolism-related polymorphisms in all subjects (*N* = 43).

Gene/Polymorphism	Genotypes			Alleles		H-W
PLIN1/rs1052700	AA: 47% (20)	AT: 42% (18)	TT: 12% (5)	A: 0.674 (58)	T: 0.326 (28)	*χ*2 = 0.094, 1 *df*, *p* = 0.759
PLIN1/rs2304795	AA: 44% (19)	AT: 44% (19)	TT: 12% (5)	A: 0.663 (57)	T: 0.337 (29)	*χ*^2^ = 0.005, 1 *df*, *p* = 0.939
LPL/rs283	CC: 63% (27)	CT: 37% (16)	TT: 0% (0)	C: 0.814 (70)	T: 0.186 (16)	*χ*^2^ = 2.247, 1 *df*, *p* = 0.134
ADRB3/rs4994	AA: 72% (31)	AG: 21% (9)	GG: 7% (3)	A: 0.826 (71)	G: 0.174 (15)	*χ*^2^ = 3.210 1 *df*, *p* = 0.073

H-W, Hardy–Weinberg equilibrium. Number in parenthesis indicates number of individuals from the total sample studied (*N* = 43).

An association between the distribution of *PLIN1* rs1052700 genotypes polymorphism with LoRes and HiRes groups was found (*χ*^2^ = 8.138; 2 *df*; *p* = 0.01; Table 4). The OR was 2.8 [95%CI 0.93–8.45] for being LoRes among A allele carriers. However, when performing an analysis with a genetic dominance model, comparing AA+AT vs. TT genotypes, we found significant differences between groups evaluated with a Fisher’s exact test (*p* = 0.012) and an RR of 1.571 [95%CI 1.57–1.63], which could be interpreted as an increased relative risk of subjects carrying AA or AT genotypes to be classified as “low responders” for their post-intervention fat mass reduction. On the other hand, no associations in the distribution of genotypes or allele frequency for *PLIN1* rs2304795, *LPL* rs283, or *ADRB3* rs4994 polymorphisms were found (Table 4).

**Table 4:** Genotype distribution and relative allelic frequencies for studied lipid metabolism-related polymorphisms by low and HiRes to fat mass reduction after the 12-week intervention (*N* = 30).

Gene/Polymorphism	Genotype and Allele	Low Responders (*N* = 19)	High Responders (*N* = 11)	*P*-value
PLIN1/rs1052700 ^*^	A/A A/T T/T	47% (9) 53% (10) 0% (0)	36% (4) 28% (3) 36% (4)	*χ*^2^ = 8.138; 2 *df*; *p* = 0.01^*^
	A T	0.737 (28) 0.263 (10)	0.500 (11) 0.500 (11)	*χ*^2^ = 3.436; 1 *df*; *p* = 0.063
PLIN1/rs2304795	A/A A/G G/G	42% (8) 37% (7) 21% (4)	36% (4) 64% (7) 0% (0)	*χ*^2^ = 3.445; 2 *df*; *p* = 0.179
	A G	0.605 (23) 0.395 (15)	0.682 (15) 0.318 (7)	*χ*^2^ = 0.352; 1 *df*; *p* = 0.553
LPL/rs283	C/C C/T T/T	63% (12) 37% (7) 0% (0)	73% (8) 27% (3) 0% (0)	*χ*^2^ = 0.287; 1 *df*; *p* = 0.592
	C T	0.816 (31) 0.184 (7)	0.864 (19) 0.136 (3)	χ^2^ = 0.229; 1 *df*; *p* = 0.632
ADRB3/rs4994	A/A A/G G/G	79% (15) 16% (3) 5% (1)	82% (9) 18% (2) 0% (0)	*χ*^2^ = 0.61; 2 *df*; *p* = 0.737
	A G	0.868 (33) 0.132 (5)	0.909 (20) 0.091 (2)	*χ*^2^ = 0.224; 1 *df*; *p* = 0.636

* genotype distribution differences.

PLIN1, perilipin 1 protein coding gene; ADRB3, adrenoceptor beta 3 protein coding gene; LPL, lipoprotein lipase protein coding gene; and df, degree of freedom. Number in parenthesis indicates number of individuals from by low- and high-responders’ groups. Bold values denote significant differences.

Following the 12-week intervention, carriers of the TT genotype of the rs1052700 variant of the *PLIN1* gene, compared to AA and AT genotypes, showed a greater reduction in absolute fat mass (Δ: − 5.1 ± 1.8 vs. − 1.8 ± 1.4 vs. − 2.1 ± 2.3 kg; *p* = 0.04; Table 5). The effect size of this fat mass reduction between TT vs. AT genotypes was a mean difference of −3.01 kg [95%IC − 4.88– − 1.1], and when comparing the TT vs. AA genotypes, the effect size was −3.29 kg [95%IC − 4.86– − 1.65]. No differences were found in modifications of body mass, BMI, FFM, maximal oxygen consumption (VO_2_peak), or VO_2_peak relative to FFM (VO_2_peak/FFM) when comparing the different genotypes of the rs1052700 variant of the *PLIN1* gene (Table 5). Finally, no differences were found in the modifications of the variables studied after the intervention when comparing the genotypes of the *PLIN1* rs2304795, *LPL* rs283, and *ADRB3* rs4994 polymorphisms.

**Table 5:** Body composition and cardiorespiratory fitness deltas after 12-week exercise intervention according to genotypes for rs1052700 PLIN1 gene polymorphism.

	Genotype AA (*N* = 13)	Genotype AT (*N* = 13)	Genotype TT (*N* = 4)	*P*-value
Δ Body mass (kg)	−1.9 ± 2.1	−1.9 ± 2.4	−5.4 ± 3.1^*^^,^^#^	0.07
Δ Fat mass (kg)	−1.8 ± 1.4	−2.1 ± 2.3	−5.1 ± 1.8^*^^,^^#^	**0.04**
Δ Body fat (%)	−1.6 ± 1.4	−1.7 ± 1.7	−4.1 ± 1.6^*^^,^^#^	0.05
Δ BMI (kg/m^2^)	−0.8 ± 0.7	−0.7 ± 0.9	−2.1 ± 1.2^*^^,^^#^	0.06
Δ FFM (kg)	−0.1 ± 1.3	+0.2 ± 1.1	−0.3 ± 1.7	0.76
Δ VO2peak (ml/min)	231.3 ± 197.5	335.9 ± 240	360.3 ± 187.2	0.39
Δ VO2peak/FFM (ml/kg/min)	5.3 ± 4.8	6.9 ± 5.1	8.2 ± 3.2	0.46

* Difference between TT and AA genotypes evaluated with Mann-Whitney U test.

# Difference between TT and AT genotypes evaluated with Mann-Whitney U test.

Data are presented as mean ± *SD*. Δ, magnitude of change after the intervention; BMI, body mass index; and FFM, fat-free mass. Bold values denote significant differences evaluated with the Kruskal-Wallis test.

## Discussion

We evaluated the association of *PLIN1* (rs1052700 and rs2304795), *LPL* (rs283), and *ADRB3* (rs4994) polymorphisms with high and LoRes to fat mass reduction after 12 weeks of HIIT and dietary energy restriction in overweight/obese adult women. Additionally, we examined whether body composition changes are influenced by these polymorphisms.

In our study, the LoRes and HiRes groups had multiple improvements in body composition and cardiorespiratory fitness after 12 weeks of intervention, where both groups decreased their fat mass, % body fat, body mass, and BMI (Table 1). In addition, they increased their absolute VO_2_peak and VO_2_peak relative to lean mass (Table 1). About changes in body composition, the reductions in absolute fat mass, body mass, % body fat, and BMI, were of greater magnitude in the HiRes group (Table 1), which is consistent with the research design where the primary criterion for classification of low and HiRes was the reduction in absolute fat mass (i.e., kilograms) less than or greater than 10%, respectively. Thus, our results showed a differential absolute fat mass modification, reducing 18.3 and 3.6% for HiRes and LoRes, respectively. All these improvements are consistent with that reported in the meta-analysis by Turk et al. (2017), showing multiple benefits of HIIT compared to traditional exercise on health-related physiological parameters in adults with obesity (Turk et al., 2017), and with the systematic review and meta-analysis by Viana et al. (2019) that showed a 28.5% greater reduction in absolute fat mass (kg) with HIIT interventions compared to MICT (Viana et al., 2019). In addition, recent systematic reviews and meta-analyses have shown more significant benefits in reducing fat mass and controlling body mass with interventions that combine exercise and nutrition (Johns et al., 2014; Clark, 2015), which is consistent with our results.

Regarding the improvements in VO_2_max and exercise performance variables during the incremental exercise test in both LoRes and HiRes groups (Table 2), this is consistent with previously published studies, where endurance performance has improved in women after a similar 16-week HIIT program (Alvarez et al., 2018). There is strong evidence demonstrating increases in VO_2_max following HIIT interventions (Gibala et al., 2012; Milanović et al., 2015), which is corroborated by the findings in our cohort of obese women. Clark et al. (2019) reported increases in VO_2_max following a 6-week HIIT intervention in obese women but showed no changes in peak O_2_ pulse, PPO, AT, RER, or VE (Clark et al., 2019). However, it should be considered that being a short duration program may not be sufficient to achieve these exercise-associated adaptations.

About cardiovascular changes, the reductions in blood pressure in both groups (Table 2), independent of being non-hypertensive subjects, are consistent with what has been previously reported in older women following 10-week resistance training (Nascimento et al., 2018) but inconsistent with other studies showing changes only in the group of hypertensive subjects following similar HIIT interventions (Olea et al., 2017; Álvarez et al., 2018). Therefore, we could hypothesize that this decrease in blood pressure values in normotensive women occurs due to the reduction of their fat mass, as there are studies that show the association between higher adiposity and increased blood pressure levels in different populations (Malden et al., 2019).

The genotypes distribution and relative frequency of alleles found for the *PLIN1* rs1052700, *PLIN1* rs2304795, *LPL* rs283, and *ADRB3* rs4994 polymorphisms (Table 3), to the best of our knowledge, are the first reports in the Chilean population. It is noteworthy that an association was found in the distribution of genotypes of the rs1052700 polymorphism of the *PLIN1* gene with the LoRes and HiRes groups, wherein the LoRes group there was no presence of the TT genotype (Table 4). Previously, the *PLIN1* rs1052700 polymorphism has been associated with body fat and waist circumference in Caucasian women but not men (Qi et al., 2004). Additionally, this same polymorphism was associated with T2D but not obesity in the Iranian population (Saravani et al., 2017) and increased risk of diabetes in Chinese adults with elevated waist circumference (Yu et al., 2013).

Among the most remarkable findings of the present study, we have the greater reduction in absolute fat mass shown by the carriers of the TT genotype of the rs1052700 variant of the *PLIN1* gene compared to the AA and AT genotypes (Table 5). These results are concordant with that found by Aller et al. (2017), where they reported an association between the TT genotype of the *PLIN1* rs1052700 polymorphism and the reduction of body mass ≥5% following 3 months of a multidisciplinary intervention with dietary advice, psychological counseling, and increased physical activity in a Caucasian obese population (Aller et al., 2017). In addition, a haplotype of two SNPs of the *PLIN1* gene, 13041A>G (rs2304795) and 14995A>T (rs1052700), has previously been associated with obesity risk (Qi et al., 2004) and with response to a 6-month endurance exercise intervention (Jenkins et al., 2010). The *PLIN1* 13041A/14995A haplotype which is present in ~ 50–55% of the white Caucasian population and has been associated with a better cardiorespiratory phenotype, body composition, and metabolism before and after an aerobic exercise intervention in Caucasian subjects compared to non-AA haplotype subjects (Jenkins et al., 2010). The Jenkins et al. (2010) study shows that a 6-month endurance exercise intervention improved body composition in AA and non-AA haplotype groups, but the elevated fat mass was maintained in non-AA carriers after training (Jenkins et al., 2010). Conversely, in our study, the highest magnitude of response to reduce absolute fat mass had by subjects of the TT genotype. It was also observed that, although no significant differences, there is a tendency to find greater reductions in body mass, % body fat, and BMI in the TT genotype (Table 5), which should be explored in larger sample sizes.

Finally, we know that the different isoforms of the perilipin are proteins that coat lipid droplets and regulate intracellular lipolysis (Bickel et al., 2009). Their phosphorylation state regulates access to triglycerides (TG) in the lipid droplet core by lipases, such as hormone-sensitive lipase and adipose triglyceride lipase, where TG hydrolysis increases with perilipin phosphorylation (Brasaemle et al., 2000). Therefore, we could hypothesize that this higher response of subjects with TT genotype could be due to a differential effect of this genotype on the phosphorylation/dephosphorylation state of perilipin. To confirm this hypothesis, future mechanistic studies that take into account these genotypes are required.

The strengths of our study include the rigorous design of the exercise protocol monitored individually on a series-by-series and session-by-session basis during the 12-week program and the individually prescribed home hypocaloric diet. Another strength was using real-time quantitative PCR for SNP genotyping. On the other hand, one of its main limitations was the small sample size, which prevents us from presenting more solid conclusions. However, this did not restrict the identification of differential response to fat mass reduction after the 12-week intervention according to *PLIN1* rs1052700 genotypes. Therefore, our preliminary data should be interpreted in light of the limited cohort evaluated. Other limitations were the lack of a non-intervention control group and the use of a BIA to measure body composition variables, as this is not considered the “gold standard” method. However, BIA has a demonstrated reliability which is crucial for the pre-post comparisons.

In conclusion, these results suggest that the rs1052700 (14995A>T) polymorphism of *PLIN1* is associated with a differential response to fat mass reduction after a 12-week intervention in overweight/obese adult women. In addition, women with the TT genotype of this genetic variant showed greater changes in fat mass than AA and AT genotypes. However, further studies are needed to confirm these findings.

## Data Availability Statement

The raw data supporting the conclusions of this article are available from the corresponding author upon reasonable request.

## Ethics Statement

The studies involving human participants were reviewed and approved by Scientific Ethics Committee, Universidad de La Frontera, Temuco, Chile. The patients/participants provided their written informed consent to participate in this study.

## Author Contributions

All authors have read the manuscript and agreed with the content. OA-M, ED, and LS conceived and designed the study. OA-M performed the intervention and experiments and wrote the paper. OA-M and ED analyzed the data. ED and LS contributed the reagents/materials and analysis tools and reviewed and edited the manuscript.

## Conflict of Interest

The authors declare that the research was conducted in the absence of any commercial or financial relationships that could be construed as a potential conflict of interest.

## Publisher’s Note

All claims expressed in this article are solely those of the authors and do not necessarily represent those of their affiliated organizations, or those of the publisher, the editors and the reviewers. Any product that may be evaluated in this article, or claim that may be made by its manufacturer, is not guaranteed or endorsed by the publisher.
